# Evaluation of astaxanthin stability under varying temperatures and ultraviolet irradiation durations based on Raman spectroscopy

**DOI:** 10.1016/j.fochx.2024.101947

**Published:** 2024-11-02

**Authors:** Xiaodan Liu, Wenjing Li, Zhiheng Yue, Jiangjin Qian, Wenjing Zhu, Huang Dai, Jiahua Wang, Fuwei Pi

**Affiliations:** a College of Food Science and Engineering, Wuhan Polytechnic University, Wuhan 430023, Hubei, People's Republic of China; b School of Food Science and Technology, Jiangnan University, Wuxi 214122, Jiangsu, People's Republic of China

**Keywords:** Astaxanthin, Confocal Raman spectroscopy, Temperature, Ultraviolet irradiation, Degradation

## Abstract

As a potent naturally carotenoid, Astaxanthin (AST) is commonly used as a natural coloring agent and antioxidant in food products, and it's stability is of great interest. The stability of AST solution stored in glass bottle under different temperatures and ultraviolet (UV) irradiation durations was analyzed in situ using confocal Raman spectroscopy, and the acceptable depth of focus was optimized. Raman spectra of AST geometrical isomers were determined by density functional theory (DFT) simulation, and characteristic peaks were selected for studying AST degradation and isomerization. Raman spectra and peak-fitting spectra based on gaussian multi-peak fitting analysis combined with Pearson's correlation analysis were conducted to study the effect of temperatures and UV irradiation on AST degradation and isomerization. The peak intensity ratio of I_1518_/I_880_ had been selected as the optimal Raman spectral variable for AST degradation based on Pearson's correlation analysis. Finally, degradation kinetic curves and degradation rate prediction equation were established. The results indicated that the isomerization of 9,13-di-cis isomer occurred at a UV irradiation of 288 h. Moreover, high temperatures above 60 °C and prolonged UV exposure exceeds 48 h can cause significant degradation of AST, with a degradation rate above 20 %. This study provided an in-situ, nondestructive potential method for the calculation of AST degradation under different temperatures and UV irradiation durations, which contribute guiding insights into the development and utilization of AST in food industry.

## Highlights

• Various isomers of astaxanthin (AST) can be distinguished by Raman spectroscopy.
• In situ stability analysis of AST by Raman spectroscopy.
• High temperature, and ultraviolet irradiation caused AST degradation.
• Prolonged ultraviolet irradiation caused the isomerization of 9,13-di-cis isomer.

## Graphical abstract

**Figure:**
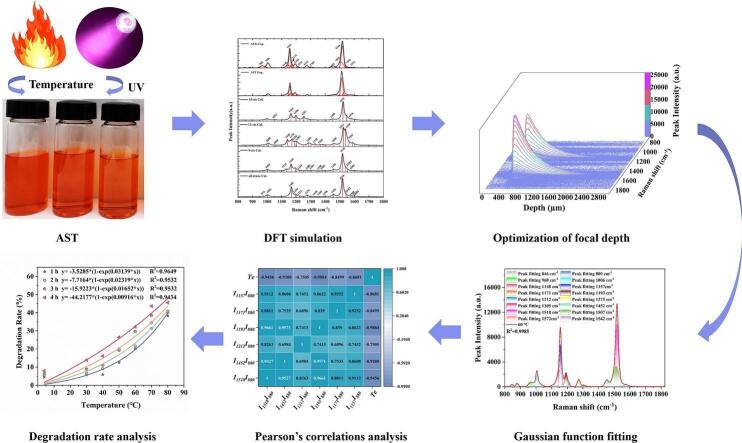

## 1 Introduction

Astaxanthin, a natural fat-soluble carotenoid, is widely found in various algae, fungi and crustacean shells. The unique molecular architecture of four isoprenoid units linked through conjugated double bonds, with a six-membered ring at each end endows astaxanthin with superior antioxidant activity, which is 10 times more potent than beta-carotene, lutein, and zeaxanthin, and 100 times more effective than vitamin E (Nair et al., 2023). Additionally, astaxanthin also exhibits significant hypoglycemic effects, prevention function of cardiovascular diseases, immune modulation ability, anti-inflammatory properties, and anticancer activities (Wu et al., 2015). The superior bioactivity and pronounced physiological functions of astaxanthin confer it with high economic value, leading to its widespread application in the food, cosmetics, aquaculture, and pharmaceuticals industries (Lima et al., 2021; Sun, 2020). Currently, global health product companies have introduced a variety of health products containing astaxanthin, such as oral liquids, soft and hard capsules (Lorenz & Cysewski, 2000). However, the presence of conjugated unsaturated double bonds and functional groups such as carbonyl and hydroxyl moieties makes it susceptible to isomerization or degradation under the inevitable influences of UV irradiation and temperature during processing, transportation, and storage, thereby affecting its physiological functions and further application (X. Liu et al., 2023). Moreover，it has been proved that astaxanthin has all-trans and nine cis isomers with different biological activities (Yao et al., 2022).

In order to improve the utilization of Astaxanthin, different steady-state encapsulation techniques have been studied based on emulsions, nanoparticles, microcapsules, and liposomes(Dang et al., 2024). Specifically, emulsion encapsulation traps active ingredients within a lotion system composed of two or more immiscible liquids (Yang et al., 2021), while nano-encapsulation confines bioactive materials at the nanoscale (C. Liu et al., 2019.). Microencapsulation involves encapsulating sensitive or active substances within polymer capsules (Cui et al., 2025), and liposome encapsulation sequesters materials within phospholipid bilayer vesicles (Wu et al., 2023). These techniques protect sensitive components including astaxanthin from external environmental factors such as light, oxygen, and temperature, thereby enhancing their stability. It's worth noted that the effectiveness of these methods is assessed based on a clear and specific understanding of the degradation and isomerization of astaxanthin under varying temperature and UV irradiation conditions. Therefore, a thorough investigation and an effective evaluation method of the impact of temperature and UV irradiation on the stability of astaxanthin are of great significance for the development and utilization of this valuable resource.

Currently, the standard methods for studying degradation and isomer transformation of astaxanthin are High-Performance Liquid Chromatography (HPLC), Ultraviolet-Visible (UV–Vis) spectroscopy, and Nuclear Magnetic Resonance (NMR) spectroscopy (Yu & Liu, 2020; Yang et al., 2017). These methods are costly and time-intensive since the necessary of skilled operators and complex pretreatment, which are also not environmentally sustainable due to reliance on chemical reagents. Besides, Fourier-transform infrared is a technique based on molecular vibrational and rotational transitions, which can also be employed for the analysis of the degradation of astaxanthin in theory. However, when analyzing aqueous solutions or samples with high water content, the strong absorption of infrared light by water molecules will interfere with the target signal and affect the analysis accuracy (Fomina et al., 2023). Raman spectroscopy provides detailed insights into molecular structure by detecting changes in molecular vibrational modes, and it is less sensitive to water molecules and therefore suffers less interference when analyzing aqueous solutions or aqueous samples (Yan et al., 2023). In particular, confocal Raman spectroscopy, with its high spatial and spectral resolution, enables in situ monitoring of isomerization and degradation at the microscale. Duan et al. applied Raman spectroscopy to analyze the stability of blueberry anthocyanins (BA) under perturbations in temperature, UV, pH, and the chemical process and mechanism of BA degradation were successfully clarified for the first time (Duan et al., 2024). Tao et al. explored the degradation of all-trans-*β*-carotene during in-vitro digestion using Raman spectroscopy, and the results revealed the degradation products of *β*-ionone and *β*-apo-carotenal (Tao et al., 2021). Novikov et al. investigated the isomerization of *β*-carotene by Raman spectroscopy, and the results indicated the positions and intensities of the CAC and C@C stretching bands are the key factors for the determination of isomer type (Novikov et al., 2021). However, there is no report on the investigation of the stability of astaxanthin with UV exposure and different temperature using Raman spectroscopy.

This study mainly explored the feasibility of using Raman spectroscopy to evaluate the stability of AST under different temperatures and UV irradiation conditions. Raman spectra of AST geometrical isomers were firstly obtained by density functional theory (DFT) simulation and calculation, and characteristic spectra were selected for studying AST degradation and isomerization. Raman spectra analysis of AST treated with different temperature and UV irradiation were then analyzed to preliminarily explore the effect of temperatures and UV irradiation on AST. Gaussian multi-peak fitting of Raman spectra of AST treated with different temperatures and UV exposure times were conducted to further analyze the isomerization and degradation, and the Pearson's correlation analysis were carried out to verify the reliability of the selected characteristic peaks for the study of AST degradation and isomerization and select the optimal Raman spectral variable for degradation kinetics and degradation rate studies. Finally, degradation kinetic curves and degradation rate prediction equation based on the optimal Raman spectral variable were established to provide a reference for development and utilization of AST.

## 2 Materials and methods

### 2.1 Sample preparation

AST standard reference material (SML0982 SIGMA, purity ≥98 %) used in this study was purchased from Solarbio (Shanghai，China). The standard AST was dissolved in the mixed solution of ethyl acetate and ethanol with a ratio of 1:1, and then sonicated for 5 min using an ultrasonic cell disruptor. The concentration of AST was 20 mg/l, and the prepared AST solution (AST_S_) were kept at −20 °C in glass bottle protected from light.

#### 2.1.1 AST_S_ treated with different temperatures

The thermal degradation test was carried out in a constant temperature controlled with water bath. In order to investigate the thermal degradation at 4 °C and at different temperatures between 30 °C and 80 °C. The samples of each test were retained for a maximum of 4 h. At predetermined time intervals of 1, 2, 3, and 4 h, the Raman spectrum of each ASTs sample was measured three times independently, and the average spectrum was utilized for follow-up analysis.

#### 2.1.2 ASTs treated with UV irradiation

ASTs was irradiated in a dark box, and the illumination condition was controlled by 10 W UV lamp (Zoomlion UV photometer) with a wavelength of 395 nm and ASTs. During the irradiation period, the samples were taken out every 24 h to measure their Raman spectra until the color of ASTs faded (Duan et al., 2024), and the final irradiation time was 312 h. Each test was conducted in triplicate, and the average spectra were used for further analysis.

### 2.2 In situ Raman measurement of ASTs in a glass bottle

To accurately measure the Raman spectra of ASTs in a glass bottle in situ, the acceptable focal depth range for the laser confocal Raman spectrometer was firstly optimized. A depth-series scan was performed, referencing the horizontally placed glass bottle's depth, with the initial measurement point set 50 μm above the bottle surface. The positive direction was defined as perpendicular to the plane of the bottle, and the total traversal distance was 2990 μm, with a step size of 10 μm.

After the determination of acceptable depth of focus, the Raman spectrum of ASTs was measured by a laser confocal micro-Raman spectrometer (model inVia Qontor; Renishaw Co., London, UK), equipped with a 532-nm laser (rated power was ca. 50 mW), a 1800 l/mm diffraction grating and a cooled charge coupled device detector. A 50 × long working distance objective and a 10 × eyepiece lens was used. The Raman spectral data in the range of 800 to 1824 cm^−1^ was recorded for two cumulative acquisitions, the exposure time and the exposure power were set to 10 s and 1 %, respectively. The spectral resolution was 1 cm^−1^, and 611 spectral variables were obtained. Specifically, the glass bottle with ASTs was placed horizontally on the movable stage of the instrument. The focus point was adjusted to the optimal acceptable depth range, and the Raman spectrum was then acquired. Each ASTs sample was measured three times independently, and the averaged spectra were calculated for subsequent analysis.

### 2.3 Simulation of the Raman spectra of AST isomers based on DFT

As a powerful tool for quantum chemical simulations, Gaussian software is capable of modeling a wide range of quantum mechanical properties of molecules and materials, including molecular structures, vibrational frequencies, and spectroscopic properties (Cerón-Carrasco et al., 2009). For the simulation of the Raman spectra of AST isomers, density functional theory (DFT) calculations were conducted utilizing the Gaussian16 computational package with the B3LYP functionals. The geometric structures of AST isomers were then optimized and frequency calculations were performed based on the triple-zeta 6–31 + G(d, p) split valence-shell basis set with the vibrational frequency scaling factor of 0.9748 (Laury et al., 2012). No constraints were imposed during the optimization process, and the optimized geometric structures were free of imaginary frequency. Subsequently, the simulation of Raman spectra was carried out with a resolution of 8 cm^−1^ based on the conversion of Raman intensity and Raman activity, which can be calculated using the method reported in previous research (Yao et al., 2022).

### 2.4 Processing of Raman spectra

#### 2.4.1 Preprocessing of Raman spectra

Due to that the presence of stray light, background light, and instrumental vibration can introduce random noise and redundant signals, several preprocessing steps were conducted to enhance the quality and signal-to-noise ratio of the spectrum.

A spike calibration method was employed to eliminate the interference from random or detector-generated cosmic rays by removing the wavenumbers of low intensity after comparing the spectral intensity and counts. A baseline correction was then conducted by utilizing a polynomial fitting method, and Savitzky-Golay filtering was also used to reduce the noise in the dataset. The above Raman spectral pre-processing were performed in Wire 5.3 software (Renishaw Co., London, UK).

#### 2.4.2 Gaussian multi-peak fitting of Raman spectra

Gaussian multi-peak fitting method is a commonly used method to address peak overlap in Raman spectra, allowing for the original Raman spectrum to be decomposed into individual Gaussian peaks to accurately quantify the peak positions, widths, and intensities (H. Wang et al., 2021). Specifically, all prominent peaks within the spectrum must be firstly identified, and a suitable Gaussian function with a good fitting performance is chosen to characterize the shape of each peak. Parameters including wavelength, width intensity of each peak are estimated, and a nonlinear least-squares optimization algorithm is employed to refine these parameters, minimizing the residuals between the model and the experimental data. The coefficient of determination (*R*^2^) is used to evaluate the performance of Gaussian fitting. In this study, gaussian multi-peak fitting of Raman spectra was conducted in Origin Pro 2021 software (Origin Lab, Northampton, MA, USA), and the equation for peak fitting is as follows:

(1) $$y=y_{0}+\frac{A}{\omega \sqrt{\pi /2}}\exp\left[-2\frac{{\left(x-x_{c}\right)}^{2}}{\omega^{2}}\right]$$

where y_0_ is the baseline; *A* is the area of the sub-peak; *ω* is the full width at half peak (FWHM) of the sub-peak; and *x*_c_ is the position of the sub-peak.

### 2.5 Degradation kinetics under different temperatures and UV irradiation

To investigate the degradation process of AST under different temperatures and UV irradiation, degradation kinetics of AST was conducted, with the spectral data of AST fitted with zero-order, first-order and second-order kinetic equations, and these equations can be referred to the reference (Dewanti et al., 2022; Z. Wang et al., 2024).

To visually analyze the impact of temperature and UV irradiation on AST, the degradation rate was also calculated, and then fitted by suitable equations to obtain a degradation rate prediction equation. The degradation rate can be obtained using the following equation.

(2) $$\mathrm{DR}=\frac{Y_{n+1}-Y_{n}}{Y_{n}}\times 100\%$$

where DR is the the degradation rate; *Y*_n_ is the AST content at the *n* th processing sequence; *Y*_*n+1*_ is the AST content at the *n* *+* *1* th processing sequence. *Y* values are the intensities of the optimized characteristic Raman spectrum.

The data processing and plotting mentioned above were conducted in Origin Pro software (Origin Lab, Northampton, MA, USA).

## 3 Results and discussion

### 3.1 The geometric structures and Raman spectra analysis of AST isomers

**Fig. 1:**
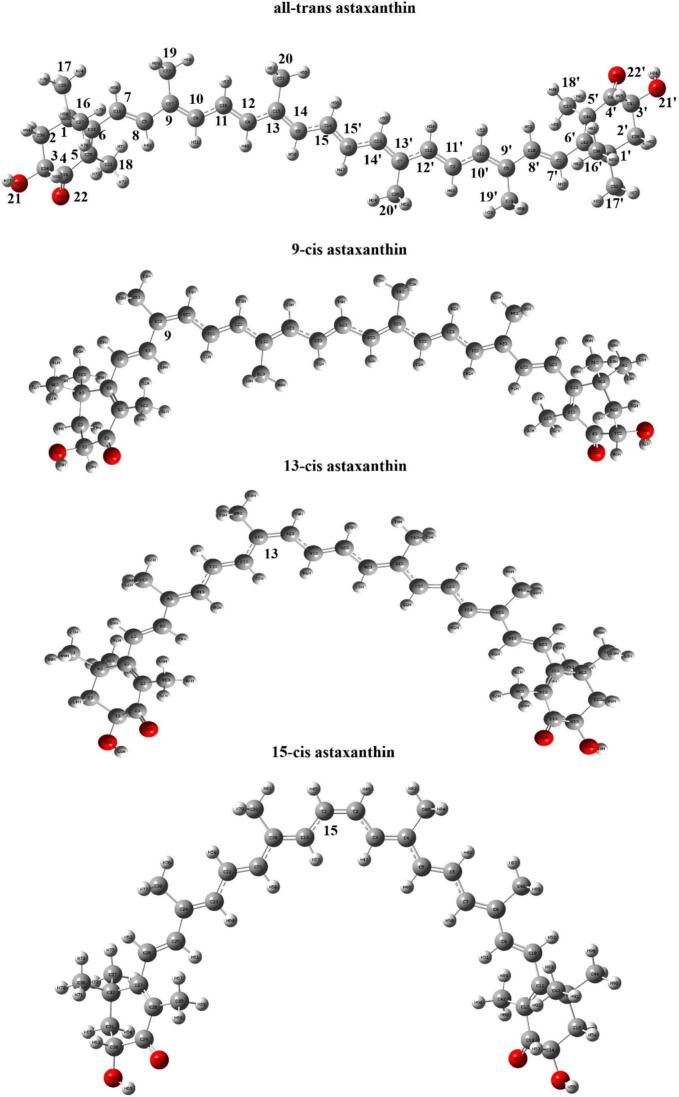
The optimized geometric structures of all-trans, 9-cis,13-cis and 15-cis isomers of astaxanthin (AST).

AST is composed of two oxidized ionone rings with a hydroxyl group (OH) at the C3 position and a carbonyl group (C=O) at the C4 position connected by a long conjugated double bond system. The extensive array of conjugated double bonds within the polyene backbone of AST results in various isomeric forms, including all-trans, 9-cis, 13-cis, 15-cis, and a di-cis isomer (Holtin et al., 2009). Among these, the all-trans isomer is the most prevalent in nature (Murakami et al., 2018). Upon exposure to high temperature and UV irradiation, AST undergoes not only degradation but also isomerization (Y. Zhang et al., 2023). The Raman characteristic peaks of AST isomers have not been differentiated in most of previous research. Consequently, to obtain the Raman spectra for the primary AST isomers, density functional theory (DFT) simulations were performed based on the structures of AST isomers, including all-trans, 9-cis, 13-cis and 15-cis AST isomers. Fig. 1 shows the optimized geometric structures of AST isomers. For the all-trans isomer, the large groups on either side of the double bonds are positioned in a mutually opposite configuration. While for the 9-cis, 13-cis and 15-cis isomers, the large groups on both sides of the double bond are oriented on the same side at the at the 9th, 13th and 15th double bond, respectively. These significant structural variations alter the vibrational patterns and Raman spectral characteristics of AST isomers.

**Fig. 2:**
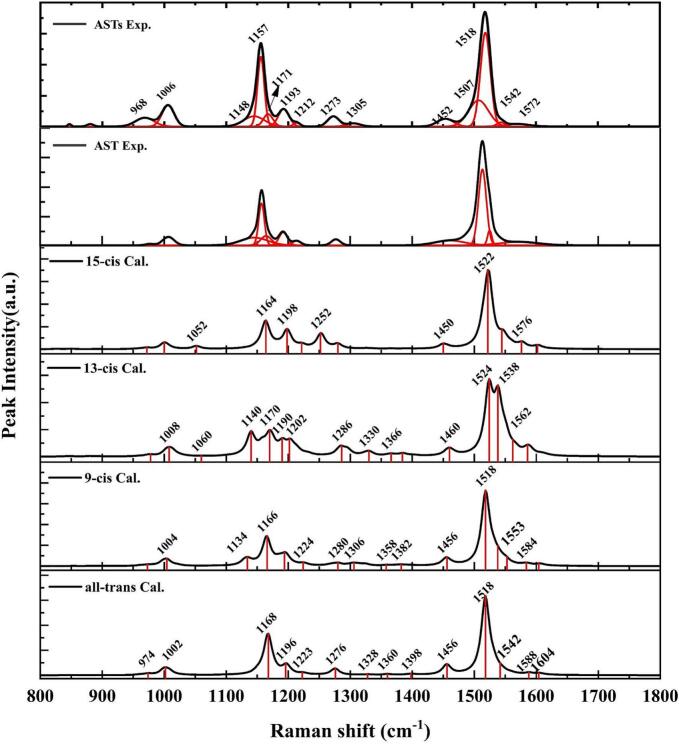
The experimental (Exp.) Raman spectra of ASTs and AST standard reference material and calculated (Cal.) Raman spectra of all-trans, 9-cis and 13-cis isomers of AST by DFT simulation. The red lines in the Exp. spectra are the fitted split peaks. The red lines in the Cal. spectra are the Raman activity intensities of the molecular vibrational modes. (For interpretation of the references to color in this figure legend, the reader is referred to the web version of this article.)

Fig. 2 shows the simulated Raman spectra of AST isomers and the experimental Raman spectra of ASTs and AST standard reference material, and the results are consistent with the previous research (Yao et al., 2022), which provides the functional group assignments corresponding to the characteristic peaks. For the Raman spectrum of all-trans isomer, several distinct characteristic peaks were observed. The weak peak at 974 cm^−1^ might be related to symmetry wagging of C8—H(11), C10—H(11), C11—H(11), C8′—H(11), C10′—H(11), and C11′—H(11). The moderate intensity band at 1002 cm^−1^ is attributed to rocking vibration of C20H3(12), C20′H3(12), C19H3(11), and C19′H3(11), and the strong band at 1168 cm^−1^ is mainly from symmetry stretching of C—C single bonds, including C14—C15, C14′—C15′, C10—C11, C10′—C11′ bonds. The band with moderate intensity at 1196 cm^−1^ is predominantly due to the stretching of C8—C9 and C8′—C9′ bonds, while the band of similar intensity at 1276 cm^−1^ is primarily associated with the rocking vibration of C11—H and C11′—H bonds. Another moderate intensity band at 1456 cm^−1^ was also obvious, which represents the scissoring vibration of C20H3(16), C20′H3(16). The most prominent band at 1518 cm^−1^ arises from the symmetry stretching of C15=C15′(10), C13=C14(11) and C13′=C14′(11). Overall, the Raman spectra of 9-cis, 13-cis and 15-cis isomers were similar to that of the all-trans isomer, with some wavenumber shifts and subtle differences observed. It should be noted that there was a characteristic band at around 1134 cm^−1^ in the spectrum of 9-cis and 13-cis, which is from the stretching of C—C single bonds, including C14—C15, C13—C20 and C14′—C15′. Its Raman activity intensity in 13-cis was 135,552, which was much higher that of 62,637 in 9-cis. It's obvious that the Raman spectrum of the three cis isomers exhibited greater similarity, with the exception of the characteristic peaks at 1170 cm^−1^, 1190 cm^−1^ and 1202 cm^−1^ in 13-cis isomer and at 1252 cm^−1^ in 15-cis isomer. The moderate intensity band at 1170 cm^−1^ is from stretching of C10—C11(43), and the weak band at 1190 cm^−1^ is from rocking vibration of C14—H(15) and stretching of C14—C15(10), while the weak band at 1202 cm^−1^ might be related to rocking vibration of C14′—H(14), C12′—H(12), and stretching of C8—C9(11). The moderate intensity band at 1252 cm^−1^ is attributed to rocking vibration of C15—H(26) and C15′—H(26), and stretching of C15=C15′(12). The weak band at 1202 cm^−1^ might be related to rocking vibration of C14′—H(14), C12′—H(12), and stretching of C8—C9(11). Besides, the shoulder band at around 1540 cm^−1^ in the spectra of all isomers was observed, which represents the asymmetric stretching of carbon‑carbon double bonds, including C15=C15′(17), C11′=C11′(16) and C11′=C12′(11). The shoulder band at around 1584 cm^−1^ in the spectra of all isomers is related to stretching of C9=C10(19) and C9′=C10′(19). In addition, the experimental Raman spectra of AST powder and solution were similar to that of the simulated Raman spectra of AST isomers, with some wavenumber shifts, and a total of 14 characteristic peaks were observed, including 968 cm^−1^ (974 cm^−1^ in simulation), 1006 cm^−1^ (1002 cm^−1^ in simulation), 1148 cm^−1^ (1134/1140 cm^−1^ in simulation), 1157 cm^−11^ (1168 cm^−1^ in simulation), 1171 cm^−1^ (1170 cm^−1^ in simulation), 1193 cm^−1^ (1190/1196 cm^−1^ in simulation), 1212 cm^−1^ (1202/1223/1224 cm^−1^ in simulation), 1273 cm^−1^ (1276 /1280/1286 cm^−1^ in simulation), 1305 cm^−1^, 1453 cm^−1^ (1456 cm^−1^ in simulation), 1507 cm^−1^, 1518 cm^−1^ (1518 cm^−1^ in simulation), 1542 cm^−1^ (1540 cm^−1^ in simulation) and 1572 cm^−1^ (1584 cm^−1^ in simulation). The assignments of 11 characteristic peaks corresponding to that of isomers based on DFT simulation have been given above. The remained characteristic peak of 1305 cm^−1^ might be related to rocking vibration of C12—H(18) and C15—H(14) in 9, 13-di-cis isomer, and the peak of 1507 cm^−1^ might be related to the stretching of C=C (Jehlička et al., 2019). The above spectral anslysis results indicated the existence of various isomers in AST samples. Besides, it's evident that the Raman spectra of AST standard reference material and ASTs were similar, which indicated that the ethyl acetate and ethanol solution commonly used in AST extraction has no significant effect on AST molecular structure.

### 3.2 Determination of an acceptable depth of focus

**Fig. 3:**
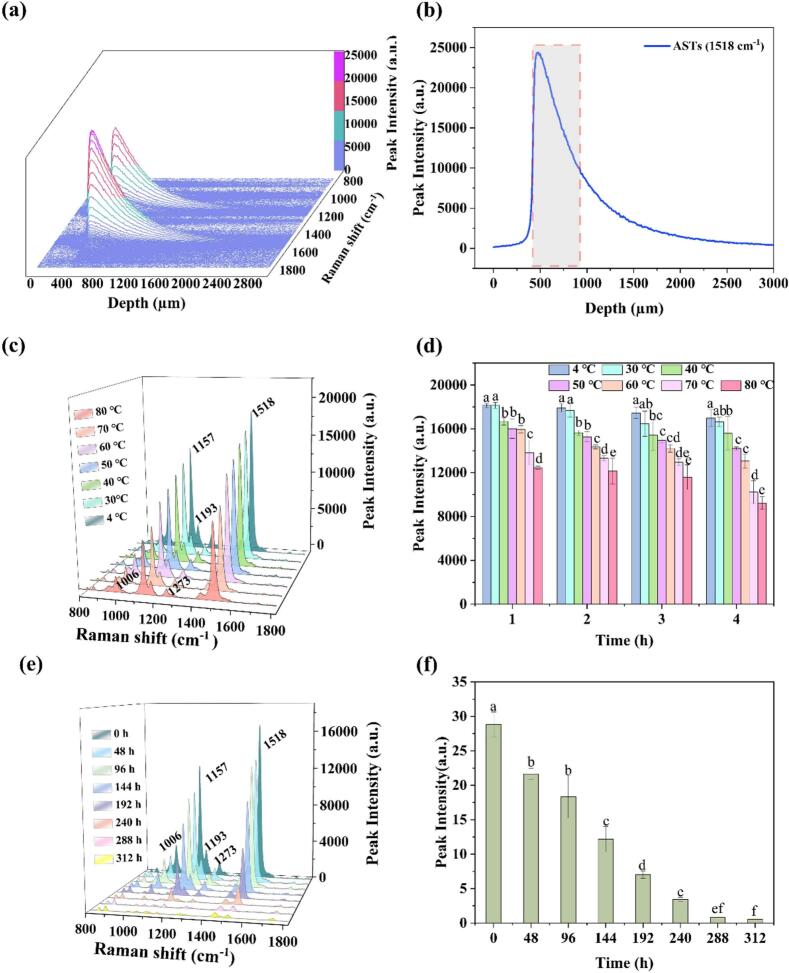
Confocal Raman spectra at different depths and intensity trends of characteristic peaks of AST packed in a glass bottle (a and b); Raman spectra of ASTs and significance analysis of the peak intensity at 1518 cm^−1^ under different temperature for 4 h (c and d); Raman spectra of ASTs and significance analysis of the peak intensity at 1518 cm^−1^ under different UV irradiation durations (e and f).

To simulate practical applications, ASTs was placed in a glass bottle for Raman analysis. Glass bottles have excellent stability and reusability, making them widely used in food packaging (Alamri et al., 2021). A deep profile analysis was then conducted to determine the acceptable focal depth of the confocal Raman spectrometer. The confocal Raman spectra of glass bottles with ASTs in the deep series are shown in Fig. 3(a), and no obvious characteristic peaks were observed on this side of the glass. As the depth surpassed 350 um, the characteristic of Raman spectra predominantly originated from AST, and the intensity increased significantly at first, followed by a gradual decrease with the increase of depth. The characteristic peak of AST at 1518 cm^−1^ was then selected to ascertain the optimal depth range, with the corresponding intensities at various depths depicted in Fig. 3(b). The characteristic Raman peak of AST could be observed when the depth exceeded 410 μm, and its intensity exhibited a maximum at approximately 470 μm and then underwent a gradual decrease with the increase of depth. At the depth of 930 μm, the intensity fell below 10,000 units, leading a lower signal-to-noise ratio. Consequently, the depth range deemed suitable for analysis was set to 410–930 μm.

### 3.3 Raman spectra analysis of ASTs treated with different temperature and UV irradiation durations

Fig. 3(c) shows Raman spectra of ASTs treated with different temperature for 4 h. It's obvious that the spectral intensity of the characteristic peaks of ASTs gradually decreased with the increase of temperature, demonstrating that high-temperature treatment led to the degradation of ASTs, which was consistent with the previous research (Silva et al., 2024). To further clarify the effect of temperature on ASTs, a significance analysis was conducted on the peak intensities of the main characteristic Raman peak at 1518 cm^−1^ of AST under various temperature treatments over different time periods, as shown in Fig. 3(d). It can be seen that there was no significant difference in peak intensity at temperatures of 4 °C and 30 °C. When the temperature was higher than 40 °C, the peak intensity decreased significantly as the temperature increased. Compared with the temperature, the treatment time had less effect on the peak intensity. The overall results showed that high temperature promotes significant degradation of AST.

Fig. 3(e) illustrates Raman spectra of ASTs under varying durations of UV irradiation. It can be observed that the intensity of the main characteristic peaks of ASTs gradually decreased as the duration of UV irradiation increased. In particular, after 288 h of UV irradiation, the Raman characteristic peak spectral intensity was relatively low, indicating a lower content of AST. The significance analysis of the peak intensity at 1518 cm^−1^ was further carried out, as shown in Fig. 3(f). The intensity of the characteristic peak for AST significantly diminished after 96 h of UV irradiation, and it progressively decreased with extended UV exposure, suggesting potential complete degradation of AST at prolonged irradiation times.

### 3.4 AST degradation analysis based on Raman spectra

Due to the potential peak overlapping in Raman spectra, single-peak fitting was conducted to more accurately investigate the degradation of AST under different temperatures and UV radiation durations. The Pearson's correlation coefficients were used to assess the influence of temperature and UV exposure time on AST peak intensity and to confirm the reliability of the fitted characteristic peaks for AST degradation analysis. Besides, to mitigate the effects of spectral fluctuations resulting from the experimental collection process, a screening process based on the Pearson's correlation analysis between peak intensity ratios of the characteristic peaks of AST to the relatively stable peaks within the system and temperature, as well as UV exposure time, was employed to select the optimal peak intensity ratio, which was then used to calculate the degradation kinetic equation and the degradation rate of AST under varying temperatures and UV treatments. It's worth mentioned that the polarity of different solvents will affect the solubility and stability (Peña et al., 2006; Sachindra et al., 2006), thus, the commonly used solvents of ethanol and ethyl acetate for the extraction of AST were used (Irshad et al., 2019; Martínez et al., 2019). To ensure that the nonpolar compound AST was fully dissolved without compromising its stability significantly (Yousef AL-Tarifi et al., 2020), a mixed solvent system of ethyl acetate and ethanol in a 1:1 ratio, which had relatively low polarity, was employed (X. Zhang et al., 2022). Hence, the degradation kinetic equation and the degradation rate of AST under varying temperatures and UV treatments in this study were valid when using a 1:1 mixture of ethyl acetate and ethanol as the solvent. Nonetheless, the results can provide reference for investigating the degradation of AST in other solvents under the influence of light and temperature.

#### 3.4.1 AST degradation analysis under different temperatures

**Fig. 4:**
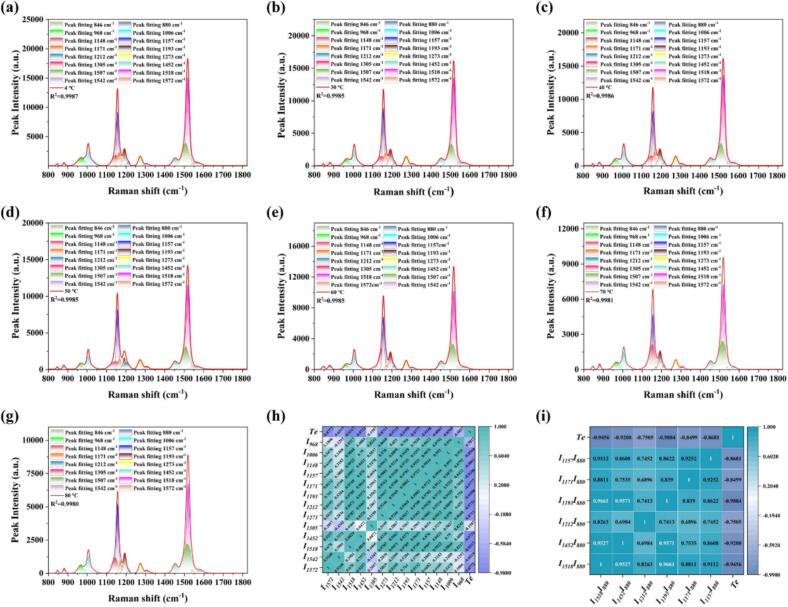
Gaussian fitting results of AST Raman spectra under different temperatures for 4 h (a–g), the results of Pearson's correlations analysis between the intensity of the characteristics peaks fitted with the Gaussian function and varying temperatures (h), and the results of Pearson's correlations analysis between the ratio of the peak intensity of AST characteristic peaks to the characteristic peak of ethanol at 880 cm^−1^ and varying temperatures (i). *T*_e_ represents temperature.

The fitted spectra of AST under different temperatures (4 °C, 30 °C, 50 °C, 60 °C, 70 °C, and 80 °C) for 4 h are provided in Fig. 4(a–g), and R^2^ were above 0.99. Characteristic peaks of ethyl acetate at 846 cm^−1^ and ethanol at 880 cm^−1^, which attributed to the C—C stretching vibration (Svensson et al., 1999) and the symmetrical stretching of the C—C—O skeleton (Emin et al., 2020), respectively, were observed, and they exhibited significant stability under different temperatures. The same 14 characteristic peaks of AST in the spectra of original ASTs samples as shown in Fig. 1 were observed. The intensities of the characteristic peaks progressively decreased with the increase of the temperature, and the peak intensity at 1518 cm^−1^ had a relative significant decrease trend, which was consistent with the change trends of original spectra. It's worth mentioned that the conjugated double bonds of AST give it an active chemical property, and the band at 1518 cm^−1^ arises from the symmetry stretching of C15=C15′(10), C13=C14(11) and C13′=C14′(11) (Yao et al., 2022). The Pearson's correlations analysis was further conducted between the intensity of the characteristics peaks fitted with the Gaussian function and the varying temperatures, as shown in Fig. 4(h). The intensities of the characteristic peaks exhibited significant negative correlation with temperature at the level of *P* ≤ 0.05, with Pearson's correlation coefficients ranging from 0.8568 to 0.9777, except that of the characteristic peaks at 968 cm^−1^, 1305 cm^−1^, 1542 cm^−1^, which showed a low response to temperature changes. As mentioned above, the band at 968 cm^−1^ is related to symmetry wagging of C8—H(11), C10—H(11), C11—H(11), C8′—H(11), C10′—H(11), and C11′—H(11) (Yao et al., 2022). The band at 1305 cm^−1^ is attributed to rocking vibration of Rocking of C8—H(21) and C12—H(19), and the band at 1542 cm^−1^ represents the asymmetric stretching of of carbon‑carbon double bonds, including C15=C15′(17), C11′=C11′(16) and C11′=C12′(11) (Yao et al., 2022). The results indicated that these chemical bonds remained unbroken during the temperature-induced degradation of AST. Significant positive correlations were observed among the eight main characteristic peaks of AST, including 1148 cm^−1^, 1157 cm^−1^, 1171 cm^−1^, 1193 cm^−1^, 1212 cm^−1^, 1273 cm^−1^, 1452 cm^−1^ and 1518 cm^−1^, with Pearson's correlation coefficients above 0.8. The above results indicated that high temperatures will destroy the specific molecular structure of AST, which were also interdependent, thus leading to the degradation of AST. Besides, the reliability of these characteristic peaks for the investigation of AST degradation had also been validated. In order to screen the optimal characteristic variable for calculating the degradation kinetic equation and the degradation rate of AST, AST characteristic peaks with a temperature correlation coefficient above 0.9 were selected, the ratio of which to the stable characteristic peak of ethanol at 880 cm^−1^ were then used for the Pearson's correlations analysis with varying temperatures, and the result was given in Fig. 4(i). The peak intensity ratio of *I*_*1518*_*/I*_*880*_ showed a significant negative correlation with temperature and positive correlation with other peak intensity ratios at the level of *P* ≤ 0.01 was then used in the calculation of AST degradation.

**Fig. 5:**
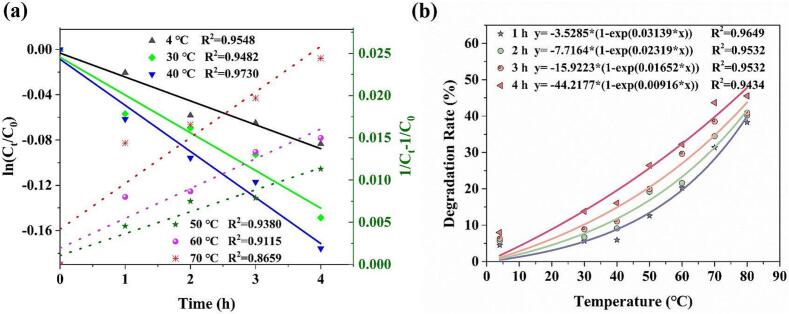
The degradation kinetics of AST at different temperatures (a), and trends in degradation rate as a function of temperature (b).

The changes of AST content characterized by the peak intensity ratio of *I*_*1518*_*/I*_*880*_ with time at different temperatures were fitted with zero-order, first-order and second-order kinetic equations, as shown in Fig. 5(a). The decrease in AST content under treatment conditions of 4 °C, 30 °C, and 40 °C adhered to a first-order kinetic equation (Fig. 5(a)), with R^2^ greater than 0.948. The data at temperatures ranging from 50 °C to 70 °C was better described by a second-order kinetic model (Fig. 5(a)), with R^2^ ranging from 0.8659 to 0.9380, respectively, which was consistent with the results of the previous research by traditional method (Dewanti et al., 2022). Upon exposure to 80 °C, the AST content exhibited a sharp decrease at 1 h, followed by minimal changes, rendering it unsuitable for fitting with kinetic equations. The kinetic equations and degradation rate constants were then calculated, which are presented in Table 1. The degradation rate constant for AST at 40 °C was higher than that at 4 and 30 °C. Furthermore, under the premise of conforming to the second-order degradation kinetic model, the degradation rate constant of AST increased with the increase of temperature, indicating that higher temperatures accelerated the degradation of AST. In order to more intuitively determine the effect of temperature on the degradation of AST, the degradation rates under different temperatures and time conditions were calculated. Moreover, an exponential equation was used to fit the changes of the AST degradation rates with temperatures at different times, and the fitted R^2^ were above 0.94, as shown in Fig. 5(b). The degradation rates of AST increased progressively with the increase of temperature and time. At temperatures of 50 °C for 1 h, the degradation rate of AST exceeded 10 %. Significant thermal degradation of AST was also observed at 70 °C and 80 °C for 1 h, with degradation rates of 31.35 % and 38.28 %, respectively, and the degradation rates reached 43.68 % and 45.54 % after 4 h. However, only a 7.99 % degradation was observed after a 4 h low-temperature (4 °C) treatment. Thus, the influence of time on the degradation of AST was relatively insignificant. Consequently, AST exhibited greater stability at lower temperatures of 4 °C, and minimizing the heating duration will result in smaller losses of AST content when high-temperature treatment is unavoidable, which provides valuable insights for the extraction, processing, and storage of AST.

**Table 1:** Degradation kinetic equations and degradation rate constants of AST at various temperatures.

Temperature (°C)	K (h^−1^)	Fitting Equation
4	0.0211 ± 0.0027	ln(C_t_/C_0_) = 0.0211x + 0.0032
30	0.0333 ± 0.0045	ln(C_t_/C_0_) = 0.0333x + 0.0069
40	0.0407 ± 0.0039	ln(C_t_/C_0_) = 0.0407x + 0.0086
50	0.0026 ± 0.0004	1/Ct-1/C0 = 0.0026x + 0.0011
60	0.0035 ± 0.0006	1/Ct-1/C0 = 0.0035x + 0.0019
70	0.0054 ± 0.0012	1/Ct-1/C0 = 0.0054x + 0.0042

K: the degradation rate constant.

#### 3.4.2 AST degradation analysis under different UV irradiation durations

**Fig. 6:**
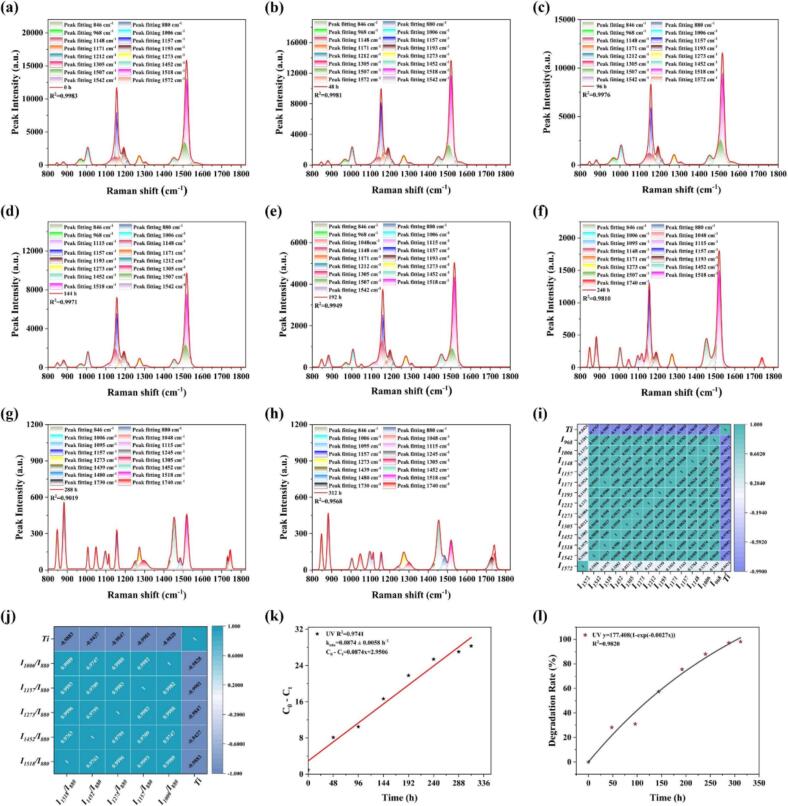
Gaussian fitting results of AST Raman spectra under different UV irradiation durations (a–h), the results of Pearson's correlations analysis between the intensity of the characteristics peaks fitted with the Gaussian function and varying UV irradiation durations (i), the results of Pearson's correlations analysis between the ratio of the peak intensity of AST characteristic peaks to the characteristic peak of ethanol at 880 cm^−1^ and varying UV irradiation durations (j), the degradation kinetics of AST at different UV irradiation durations (k), and trends in degradation rate as a function of UV irradiation durations (l). *T*_i_ represents time.

The fitted spectra of AST under varying durations of UV irradiation are provided in Fig. 6(a–h), and R^2^ was between 0.9019 and 0.9983. Characteristic peaks of ethyl acetate at 846 cm^−1^ and ethanol at 880 cm^−1^ were also detected, which were relatively stable. The same 14 characteristic peaks of AST in the spectra of original AST samples in Fig. 1 were also observed. With increasing duration of UV irradiation, the intensity of the characteristic peaks of AST progressively decreased, leading to the gradual appearance of the characteristic peaks of ethyl acetate (1048 cm^−1^, 1095 cm^−1^, 1245 cm^−1^, 1730 cm^−1^ and 1740 cm^−1^) (Adhikari & Khatri, 2021; Neelakantan, 1963.) and ethanol (1048 cm^−1^, 1095 cm^−1^, 1115 cm^−1^, 1439 cm^−1^, 1480 cm^−1^) (Burikov et al., 2010; Li, 2018) with a smaller peak intensity after 144 h of UV exposure. Among them, and the peak intensity at 1518 cm^−1^ had a relative significant decrease trend, which were consistent with the change trends of original spectra and the results of temperature-induced degradation of AST. Besides, the characteristic peak 1148 cm^−1^ representing the 9-cis and 13-cis isomers disappeared after 288 h of UV exposure, and the characteristic peak 1171 cm^−1^ and 1212 cm^−1^ representing the 13-cis isomer disappeared after 288 h and 240 h of UV exposure, respectively, indicating complete degradation of these AST isomers with the break of C14—C15, C13—C20 and C14′—C15′ reflected by 1148 cm^−1^, C10—C11(43) reflected by 1171 cm^−1^ and C14′—H(14), C12′—H(12) reflected by 1212 cm^−1^ (Yao et al., 2022). Furthermore, the representative characteristic peak 1305 cm^−1^ in 9,13-di-*cis* isomer gradually decreased and became undetectable after 200 h of UV irradiation, and its reappearance after 280 h suggested that AST underwent isomerization concurrently with degradation, which also indicated the UV irradiation-induced isomerization of 9,13-di-*cis* isomer primarily occured at C8—H(21) and C12—H(19) reflected by 1305 cm^−1^ (Yao et al., 2022). Fig. 6(i) illustrates the Pearson correlation coefficients between the Gaussian-fitted spectral intensities and UV irradiation durations. The intensities of the characteristic peaks exhibited strong negative correlations with both the UV irradiation time and each other (*P* ≤ 0.05), with coefficients exceeding 0.95, except for the peak at 1572 cm^−1^, which showed a low response to UV irradiation. The above results indicated that UV irradiation will also destroy the specific molecular structure of AST, which were also interdependent, further leading to the degradation of AST. It's also remarkable that C9=C10(19) and C9′=C10′(19) reflected by the band of 1572 cm^−1^ remained unbroken during the UV irradiation-induced degradation of AST. Besides, the reliability of these characteristic peaks for the investigation of AST degradation under UV irradiation had also been validated. Subsequently, the characteristic peaks with the Pearson's correlation coefficients above 0.95 in Fig. 6(i) that also persistently remained during the UV irradiation process were selected, and the ratio of the selected peak intensity of AST to characteristic peaks of ethanol at 880 cm^−1^ were used for the Pearson's correlations analysis with the varying UV irradiation durations, and the results were presented in Fig. 6(j). All peak intensity ratios had a significant negative correlation with UV irradiation durations, and a positive correlation with each other at the level of *P* ≤ 0.01, with the Pearson's correlation coefficients above 0.94. The peak intensity ratio of *I*_*1518*_*/I*_*880*_ that had a relative high correlation coefficient greater than 0.97 with UV irradiation durations and other peak intensity ratios was selected for the calculation of AST degradation.

Fig. 6(k) shows that the changes of AST content characterized by the peak intensity ratio of *I*_*1518*_*/I*_*880*_ with different durations of UV irradiation were well fitted with the zero-order kinetic equation, with *R*^2^ greater than 0.97. The kinetic equations and degradation rate constant are also provided. Fig. 6(l) shows the degradation rate as a function of UV irradiation time. The degradation rate of AST increased with the increase of UV irradiation time, and the AST degradation rate reached 36.33 % and 98.12 % after 48 h and 312 h of UV irradiation, respectively. Besides, a fitted exponential equation for the prediction of AST content under different UV irradiation times was obtained, with the fitted *R*^2^ above 0.98, which can also provide reference for the extraction, processing, and storage of AST.

## 4 Conclusions

In this study, stability of AST solutions in glass bottles under different temperatures and UV irradiation durations individually were in situ analyzed using confocal Raman spectroscopy. Specifically, Raman characteristic peaks of AST were identified based on DFT simulation, and 14 characteristic peaks representing different molecular structures were determined for the analysis of AST degradation and isomerization. Raman spectra and peak-fitting spectra based on gaussian multi-peak fitting analysis combined with Pearson's correlation analysis clarified the effect of different temperatures and UV irradiation durations on the stability of AST, and both temperatures and UV irradiation-induced AST degradation based on the break of C15=C15′(10), C13=C14(11) and C13′=C14′(11) accounted for the main proportion, while the isomerization of 9,13-di-cis isomer occurred at a UV irradiation of 288 h. High temperatures above 60 °C and prolonged UV exposure exceeds 48 h can cause significant degradation of AST, with a degradation rate above 20 %. Therefore, to maintain the activity of AST, the temperature should be kept below 60 °C during processing, storage, and transportation, and it should be stored away from light. Besides, degradation rate prediction equation under different temperatures and UV irradiation durations were established based on the peak intensity ratio of *I*_*1518*_*/I*_*880*_, which can provide guidance for the production, processing and preservation of AST. This study provides a new nondestructive method of confocal Raman for stability studies of AST, which enables in situ analysis of products packaged in glass bottles.

## CRediT authorship contribution statement

**Xiaodan Liu:** Writing – review & editing, Writing – original draft, Project administration, Investigation, Conceptualization. **Wenjing Li:** Writing – review & editing, Methodology, Investigation, Data curation. **Zhiheng Yue:** Writing – review & editing, Software. **Jiangjin Qian:** Writing – review & editing, Data curation. **Wenjing Zhu:** Writing – review & editing. **Huang Dai:** Writing – review & editing. **Jiahua Wang:** Writing – review & editing, Supervision, Funding acquisition, Conceptualization. **Fuwei Pi:** Writing – review & editing.

## Declaration of competing interest

The authors declare that they have no known competing financial interests or personal relationships that could have appeared to influence the work reported in this paper.

## Data Availability

Data will be made available on request.
